# Sputum signatures for invasive pulmonary aspergillosis in patients with underlying respiratory diseases (SPARED): study protocol for a prospective diagnostic trial

**DOI:** 10.1186/s12879-018-3180-z

**Published:** 2018-06-11

**Authors:** Wei Xiao, De-ying Gong, Bing Mao, Xin-miao Du, Lin-Li Cai, Min-yu Wang, Juan-juan Fu

**Affiliations:** 1 Respiratory Group, Department of Integrated Traditional Chinese and Western Medicine, West China Hospital, Sichuan University, Chengdu, China; 2 Laboratory of Anesthesia and Critical Care Medicine, Translational Neuroscience Center, West China Hospital, Sichuan University, Chengdu, China; 3 Department of Respiratory and Critical Care Medicine, West China Hospital, Sichuan University, Chengdu, China

**Keywords:** Invasive pulmonary aspergillosis, Underlying respiratory diseases, Diagnosis, Biomarker, Galactomannan, Lateral flow device, Real-time PCR, Triacetylfusarinine, Bis(methylthio)gliotoxin

## Abstract

**Background:**

Invasive pulmonary aspergillosis (IPA) has been increasingly reported in patients with underlying respiratory diseases (URD). Early diagnosis of IPA is crucial for mortality reduction and improved prognosis, yet remains difficult. Existing diagnostic tools for IPA largely rely on the detection of biomarkers based on serum or bronchoalveolar lavage fluid (BALF), both of which have their limitations. The use of sputum sample is non-invasive, and *Aspergillus* detection is feasible; however, the usefulness of sputum biomarkers for the diagnosis of IPA, especially in patients with URD, has not been systematically studied.

**Methods:**

This is a prospective diagnostic trial. At least 118 participants will be recruited from respiratory wards and intensive care units. IPA is defined according to the EORTC/MSG criteria modified for patients with URD. Induced sputum and blood will be collected, and BALF will be obtained by bronchoscopy. Sputum biomarkers, including galactomannan, *Aspergillus* DNA, triacetylfusarinine and bis(methylthio)gliotoxin will be determined, and the presence of a JF5 antigen will be examined with a lateral fluid device. The sensitivity, specificity, negative predictive value, positive predictive value and diagnostic odds ratio will be computed for different biomarkers and compared using the McNemar χ^2^ test. Receiver operating characteristic analyses will be performed, and the cut-off values will be established. Participants will receive follow-up evaluations at 3 months and 6 months after recruitment. The difference in hospital stay and survival will be analysed, and the relationships between the levels of biomarkers and hospital stay and survival will be analysed via regression models.

**Discussion:**

We have developed and verified the feasibility of *Aspergillus*-related biomarker assays for sputum. The study findings will contribute to a novel look at the diagnostic performance of sputum biomarkers in IPA and provide important insight into the improvement of the early diagnosis of IPA, particularly in patients with URD.

**Trial registration:**

This study has been registered with the Chinese Clinical Trial Registry (ChiCTR-DPD-16009070) on 24th of August 2016.

## Background

Invasive pulmonary aspergillosis (IPA) is a fungal infection that occurs primarily in patients with severe immunodeficiency [1]. In recent years, IPA has been increasingly recognized in patients without haematologic malignancy or organ transplantation [2–9], especially in those with underlying respiratory diseases (URD) [2–5, 10–12]. Remarkably, the mortality rate among non-neutropenic patients was significantly higher than that in neutropenic patients [12], such that a mortality rate of greater than 90% in patients with chronic obstructive pulmonary disease was reported [13].

Although a favourable clinical outcome of patients with IPA is largely influenced by the initiation of effective antifungal treatment [14], the early diagnosis of IPA is still notoriously difficult, and few diagnostic tools are available [15]. The “gold standard” to confirm the diagnosis is the histopathological examination of lung tissue obtained by thoracoscopic or open-lung biopsy [16]. Considering its limitation in terms of feasibility and sensitivity in clinical practice, the European Organization for Research and Treatment of Cancer/Mycoses Study Group (EORTC/MSG) proposed diagnostic criteria for IPA that integrate host factors, clinical evidence and mycological findings [17]. However, the diagnosis of IPA in patients with URD remains challenging. The host factors defined by the EORTC/MSG framework do not necessarily apply to patients in the respiratory wards or intensive care units [2], and the official group recognized this as “an omission” [17].

**Fig. 1:**
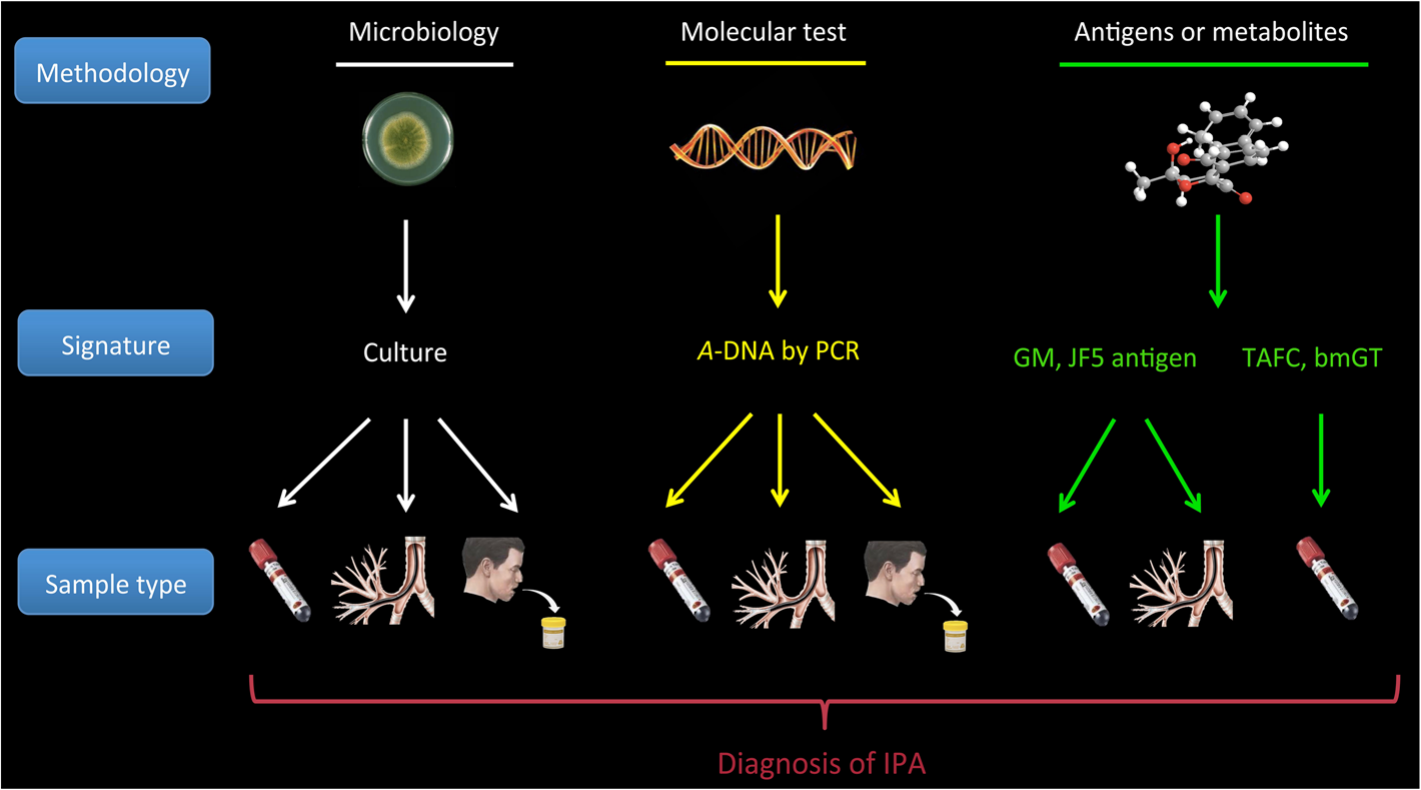
Current biomarkers for the diagnosis of IPA using different sample types. Abbreviations: *A*-DNA = aspergillus DNA; bmGT = bis(methylthio)gliotoxin; GM = galactomannan; PCR = polymerase chain reaction; TAFC = triacetylfusarinine

A variety of sample types and biomarkers have been explored and validated in the journey to seek mycological evidence for the diagnosis of IPA, and different methodologies and matching signatures have been investigated (Fig. 1). Direct mycological proof by culture is rarely feasible due to the low sensitivity and long turnaround time [18, 19]. Therefore, indirect tests that detect *Aspergillus*-related biomarkers, e.g., galactomannan (GM), in body fluids were developed, showed improved diagnostic performance and were included in the EORTC/MSG framework [17]. Serum biomarkers, in particular, *Aspergillus* DNA (*A*-DNA) and GM, have been intensively studied in haematological patients in the past two decades which exhibited good performance [20, 21]. However, these tests had lower sensitivity when applied in non-haematological patients [22, 23], probably because non-neutropenic patients tend to develop airway invasive aspergillosis instead of angioinvasive aspergillosis [24, 25]. This further indicates that biomarker detection based on organ specific samples may be associated with better diagnostic performance for IPA in non-neutropenic patients. Indeed, biomarker detection using bronchoalveolar lavage fluid (BALF) has been found to be associated with a higher diagnostic accuracy for IPA compared to serum in these patients [6–8, 26]. However, bronchoscopy is an invasive procedure and is not feasible for all patients in clinical practice. In contrast, induced sputum sampling is non-invasive, convenient and low cost and, if successful, often precludes the need for bronchoscopy. Sputum is expectorated from local sites of infection and can be used for microbiologic examinations, therefore, it has the potential to be used for biomarker determination in IPA.

We searched the literature using the terms “sputum”, “sputa”, “bronchial secretion”, “bronchial aspirate”, “respiratory sample” and “aspergillosis”, “*Aspergillus*” in Medline, EMBASE and PubMed databases up to the 20th of May 2017. Seven studies that assessed the GM assay with sputum samples were identified (Table 1), of which only the study by Kimura et al. [27] tested the efficacy of sputum GM for the diagnosis of IPA in haematological patients in a well-designed manner. The results of this study identified a sensitivity of 100% and a specificity of 62.2% for sputum GM at the cut-off value of 1.2 optical density index (ODI). However, the small sample size impeded the external validity of the study and the validation of an optimal cut-off value. Some studies have assessed *A*-DNA in sputum via the polymerase chain reaction (PCR) assay; however, these studies exclusively aimed at identifying the existence of *Aspergillus* in sputum rather than assessing the diagnostic performance for IPA [28–31]. Fraile et al. [32] and Chanzá et al. [33] intended to evaluate the sputum PCR assay for the diagnosis of IPA; however, diagnostic criteria according to the EORTC/MSG framework were not applied. Our study will use real-time PCR (RT-PCR) to detect sputum *A*-DNA for IPA in patients with URD diagnosed by the EORTC/MSG criteria.

**Table 1:** Studies assessing sputum GM assay for various *Aspergillus* events

Author	Year	*Aspergillus* events	Underlying diseases	Sputum type
Kimura [27]	2009	IPA	HD	spontaneous/induced
Fraile [32]	2012	IPA^a^	not classified^b^	not reported
Delfino [34]	2012	AC	cystic fibrosis	not reported
Baxter [29]	2013	AC	cystic fibrosis	spontaneous
Baxter [28]	2013	ABPA	cystic fibrosis	spontaneous
Chanzá [33]	2014	IPA^a^	HD and NHD	not reported
Dhillon [35]	2016	ABPA	cystic fibrosis	not reported

*Abbreviations*, *ABPA* allergic bronchopulmonary aspergillosis, *AC Aspergillus* colonization, *HD* haematological disease, *IPA* invasive pulmonary aspergillosis, *NHD* non-haematological disease

^a^EORTC/MSG diagnostic criteria were not used.

^b^Patients recruited from Infectious, Pneumology, Oncology Services and Critical Care Unit, without specific underlying diseases classified.

Several other biomarkers that either target *Aspergillus* antigens or *Aspergillus*-related metabolites have been proposed for the diagnosis of IPA in recent years (Fig. 1) by a systematic literature review. Thornton et al. [36] developed a lateral flow device (LFD) that could be used at bedside to detect the JF5 antigen, an *Aspergillus* glycoprotein secreted during active fungal growth, which showed better diagnostic performance than GM in serum and BALF. Triacetylfusarinine (TAFC) and bis(methylthio)gliotoxin (bmGT) are toxic metabolites of *Aspergillus* and have been assayed by liquid chromatography tandem mass spectrometry in serum of haematology patients [37] or neutropenic patients, respectively. The above *Aspergillus*-related signatures present appropriate diagnostic accuracy for the diagnosis of IPA, yet have not been assessed in sputum samples or in patients with URD.

A study exploring the usefulness and diagnostic performance of sputum biomarkers for IPA in patients with URD is absent. Considering the incidence and high mortality of these patients and the great potential of using a sputum sample for *Aspergillus* detection, we will conduct this study to examine a broad spectrum of approaches and biomarkers related to *Aspergillus* detection using sputum samples from patients with URD to investigate the diagnostic performance of these sputum signatures for IPA in this study population. This study may help provide novel and useful diagnostic modalities, stepping over the existing obstacles to an early diagnosis of IPA in clinical practice.

## Methods/design

### Study design

This is a prospective diagnostic trial. The study protocol has been registered with the Chinese Clinical Trial Registry (ChiCTR-DPD-16009070). Participants will be enrolled from respiratory wards and intensive care units in the West China Hospital, Sichuan University of China. Inclusion criteria are patients (1) with URD including chronic pulmonary diseases (COPD, asthma, lung cancer, bronchiectasis, interstitial lung diseases, and cystic fibrosis) and acute pulmonary diseases (bacterial pneumonia, viral pneumonia, and acute bronchitis); (2) willing to provide informed consent; and (3) able to tolerate sputum induction. Exclusion criteria are patients (1) with underlying haematological malignancies; or (2) that are previous receipts of solid organ transplant; or (3) that are unable to produce sputum.

### Diagnostic criteria

The diagnostic criteria of IPA are based on the 2008 EORTC/MSG framework [17]. Host factors, clinical criteria and mycological criteria to establish the diagnosis of IPA in this study population are listed in Table 2. In accordance with the study by Prattes et al. [11], URD is added as one of the host factors. We propose to make this modification due to several reasons. Host factors of the diagnostic criteria in previous guidelines were originally defined for haematological patients but have not been validated in non-neutropenic patients. Additionally, those typical host factors promoted by EORTC/MSG are frequently absent in non-haematological patients [2, 23]. Patients will be classified into proven, probable, possible and no IPA groups according to the revised 2008 EORTC/MSG criteria [17] (Table 3).

**Table 2:** The diagnostic framework for IPA in patients with URD

Host factors	Recent history of neutropenia (< 0.5 × 10^9^ neutrophils/L for > 10 days) temporally related to the onset of infection Prolonged use of corticosteroids at a mean minimum dose of 0.3 mg/kg/day of prednisone equivalent for > 3 weeks Treatment with immunosuppressants during the past 90 days Inherited severe immunodeficiency Underlying respiratory diseases including chronic pulmonary diseases (COPD, asthma, lung cancer, bronchiectasis, interstitial lung diseases, and cystic fibrosis) and acute pulmonary diseases (bacterial pneumonia, viral pneumonia, and acute bronchitis)
Clinical criteria	The presence of 1 of the following 3 signs on chest computed tomography: Dense, well circumscribed lesion(s) with or without a halo sign Air-crescent sign Cavity on chest computed tomography
Mycological criteria	Cytologic or microscopic evidence of *Aspergillus* elements or recovery of *Aspergillus* in sputum or BALF Positive GM test in serum or BALF

**Table 3:** IPA classification according to revised 2008 EORTC/MSG criteria

Proven IPA	Histopathological or cytopathological examination of lung tissue showing *Aspergillus* hyphae from needle aspiration or biopsy specimen with evidence of associated tissue damage, OR positive culture result for *Aspergillus* from a sample obtained by sterile procedure from the lung.
Probable IPA	Host factors, AND clinical criteria, AND mycological criteria
Possible IPA	Host factors AND clinical criteria
No IPA	Other cases excluded from proven, probable and possible IPA groups

### Participants screening and recruitment

**Fig. 2:**
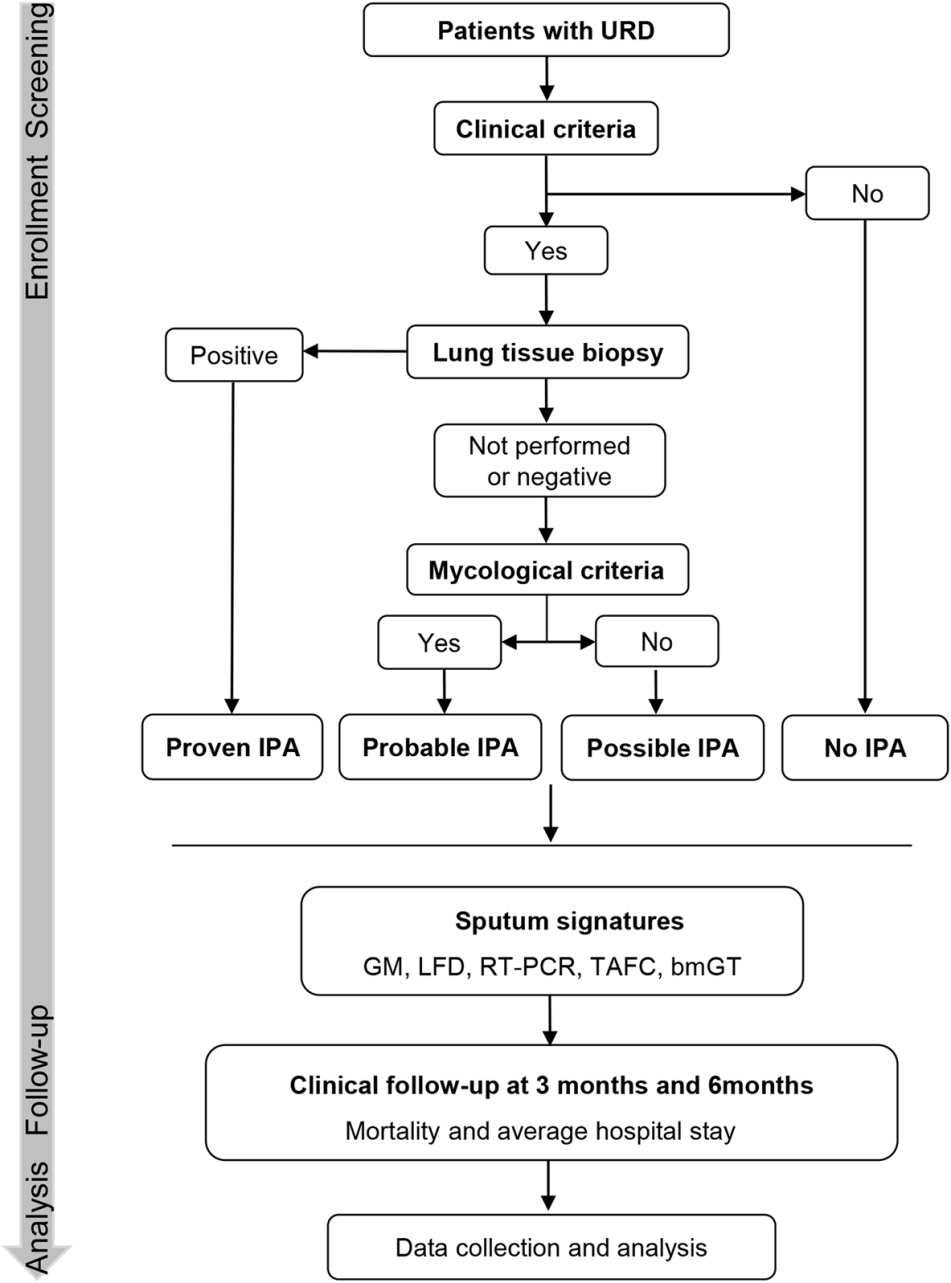
Study flowchart. Abbreviations: bmGT = bis(methylthio)gliotoxin; BALF = bronchoalveolar lavage fluid; GM = galactomannan; IPA = invasive pulmonary aspergillosis; LFD = lateral flow device; RT-PCR = real time polymerase chain reaction; TAFC = triacetylfusarinine

The study flowchart is shown in Fig. 2. Patients with respiratory diseases admitted into the hospital will be screened for inclusion and exclusion criteria for recruitment. The recruited patients will be subsequently assessed according to the clinical criteria shown in Table 2, namely newly-developed abnormalities in CT scan associated with IPA, for the classification of no IPA or suspected IPA groups. Patients without clinical criteria will be identified as no IPA. For patients with typical clinical criteria, those presenting histopathological or cytopathological evidence of *Aspergillus* hyphae from needle aspiration or biopsy specimen, or positive culture result for *Aspergillus* from a sample obtained by sterile procedure from the lung will be identified as proven IPA. If lung biopsy is not performed or negative result is generated, mycological examinations will be performed including sputum/BALF culture or serum/BALF GM tests to further classify patients as probable or possible IPA. Sample collection and testing will be required to be performed prior to antifungal treatment.

### Sputum induction and sample processing

Sputum induction using hypertonic saline (4.5%) and spirometry (KoKo PD Instrumentation, Louisville, KY) will be performed as previously described [38]. 4.5% saline will be inhaled from an ultrasonic nebulizer for doubling time periods (from 30 s to 4 mins), and a fixed cumulative sputum induction time of 15 min will be used for all participants.

Fungal culture will be performed on 100 μL of sputum plugs carefully separated from saliva and inoculated directly onto potato dextrose agar [39]. The plates will be sealed and incubated for 7 days at 37 °C. After 7 days, *Aspergillus* will be identified based on macroscopic and microscopic features.

A minimum volume of 0.5 mL of sputum plugs will be pipetted into a sterile falcon centrifuge tube, and 8 volumes of phosphate buffer solution will be added, vortexed and incubated at room temperature for 30 mins, and 1.5 mL of the above sputum suspension will be stored for *A*-DNA extraction and RT-PCR assay.

A total of 200 μL of filtered suspension will be used for quality assessment as previously described [38]. Briefly, in an adequate number of cells, the presence of pulmonary macrophages and the proportion of squamous cells are examined. A total cell count of leukocytes and cell viability is performed, and samples with a squamous cell percentage greater than 50% or viability less than 40% are excluded. The remainder of the sample will go through a sterile filter (Merk Millipore Ltd., Ireland) and will be stored in aliquots at − 80 °C for subsequent GM, LFD, TAFC and bmGT detection.

### BALF collection and sampling

Fibreoptic bronchoscopy with bronchoalveolar lavage is performed in accordance with the American Thoracic Society guidelines [40]. Briefly, the bronchoscope will be placed in a wedge position within the selected bronchopulmonary segment. A total volume of 40 mL of normal saline (at room temperature) will be divided into two aliquots and instilled through the bronchoscope. After the instillation of each aliquot, instilled saline will be retrieved with a negative suction pressure of less than 100 mmHg that can avoid visible airway collapse. The retrieved BALF (at least 5 mL) will be subdivided into aliquots within 1 h of collection after being shaken to ensure homogeneous mixing, and stored at − 80 °C for GM detection.

### GM detection

Peripheral venous blood will be collected into Vacutainer® tubes (BD Worldwide, Suzhou, China), and serum will be obtained by centrifugation at 3000 rpm, 4 °C for 10 mins. The GM assay on serum, BALF and sputum will be performed with the Platelia *Aspergillus* enzyme immunoassay (Bio-Rad, California, USA) according to the manufacturer’s instructions. Tests for serum and BALF GM will be considered positive at cut-off values of ≥0.5 ODI and ≥ 1.0 ODI, respectively.

### LFD performance

LFD will be performed as Thornton et al. described [36]. Briefly, 100 μL pretreated serum (1:1 diluted with tissue culture medium), neat BALF or processed sputum supernatant will be added to the release port on the LFD device after gentle mixing and will be incubated at room temperature for 15 mins. The development of the control line in the result window shows that the test has run correctly, and the development of the *Aspergillus*-specific test line will be determined after 15 mins. The results will be recorded as positive if the test line is present, or be recorded as negative if the test line is absent. Each LFD result will be assessed by two researchers independently.

### *A*-DNA extraction and RT-PCR

Fungal DNA will be extracted from 1.5 mL of the sputum suspension with the commercial MycXtra fungal DNA extraction kit (Myconostica Limited, Manchester, UK), and *A*-DNA will be detected with the MycAssay *Aspergillus* kit (Myconostica Limited, Manchester, UK), according to the manufacturer’s instructions. PCR runs will be performed on a CFX Connect™ RT-PCR system (Bio-Rad, California, USA) by targeting a portion of the 18S ribosomal gene, in accordance with the detection kit protocol. Ten microliters of extracted DNA will be used in a final reaction volume of 25 μL. The PCR protocol is as follows: 10 mins at 94 °C; 10 s at 94 °C, 58 s at 57 °C, and 20 s at 72 °C for 40 cycles. The cycling threshold will be recorded and used for subsequent analysis, including the determination of an optimal cut-off value, based on which the sputum samples will be regarded as positive or negative for *Aspergillus* infection.

### TAFC and bmGT detection

High-performance liquid chromatography tandem mass spectrometry (HPLC-MS/MS) on Agilent 1260–6460 (Agilent Technologies Inc., Santa Clara, USA) was first developed in our laboratory and will be used to simultaneously analyse TAFC and bmGT from sputum. The method employs phenacetin as the internal standard, and shows the lower and upper limits of quantitation for TAFC and bmGT assays of 1.56 ng/mL and 100 ng/mL, respectively. Upon detection, 100 μL of sputum will be spiked with phenacetin, vortexed and extracted with 400 μL of dichloromethane. The extraction process will be repeated once, and the total solvent will be evaporated with a pressure gas blowing concentrator. The residue will then be reconstituted in 100 μL of acetonitrile/water (50/50 *v*/v%), vortexed and centrifuged. Three microliters of supernatant will be injected for HPLC-MS/MS analysis. Standards of TAFC (EMC, Germany) and bmGT (Enzo Life Sciences, Inc., USA) are spiked into sputum samples from four patients with bacterial pneumonia to evaluate the recovery of both compounds. Each of the four sputum samples is divided into three aliquots, with one aliquot as a control and the other two spiked with different concentrations of TAFC and bmGT. We assayed one sputum sample from a patient diagnosed with lung cancer (squamous cell carcinoma) and probable IPA using the HPLC-MS/MS method.

### Clinical follow-up

Patients will receive telephone reviews at 3 months and 6 months after enrolment. Information that includes the patients’ symptoms, days of hospitalization and survival will be recorded and checked in the medical record system.

### Sample size calculation

GM is the most studied biomarker for the diagnosis of IPA among our candidate signatures; therefore, the sample size calculation is based on the diagnostic accuracy of the sputum GM assay reported by previous studies. Receiver operating characteristics (ROC) curves show the trade-off between sensitivity and specificity, and the area under the curve (AUC) is considered as an overall index of accuracy for quantitative biomarkers. The sample size estimation is based on AUC reported previously [27].

With 95% confidence (*α* = 0.05) to guarantee the marginal error of estimate (i.e. the difference between true AUC and its estimate) does not exceed from a pre-determined value of d = 0.09, and expecting an equal number of diseased and non-diseased cases, the required sample size for each group is:

1 $$ \mathrm{n}=\frac{{\mathrm{z}}_{\frac{\alpha }{2}}^2\ \mathrm{V}\left(\hat{\mathrm{AUC}}\right)}{d^2} $$

V($\widehat{\mathrm{AUC}}$) denotes the variance of $\widehat{\mathrm{AUC}}$ that is estimated parametrically based on binormal assumption [41, 42]. This binormal assumption is chosen because our study implements a four-category rating scheme (i.e. proven IPA, probable IPA, possible IPA and no IPA). $\mathrm{V}\left(\widehat{\mathrm{AUC}}\right)$ is calculated as follows:

2 $$ \mathrm{V}\left(\widehat{\mathrm{AUC}}\right)=\left(0.0099\times {e}^{\raisebox{1ex}{$-{a}^2$}\!\left/ \!\raisebox{-1ex}{$2$}\right.}\right)\times \left(6{a}^2+16\right) $$

where a = *φ*^−1^(AUC) × 1.414 and *φ*^−1^ is the inverse of standard cumulative normal distribution. With a pre-specified AUC of 0.795 according to Kimura et al. [27], the required sample size for each group is 59 after above calculation. Therefore, we will recruit a minimum number of 118 participants with 59 controls (no IPA) and 59 cases (proven or probable IPA).

### Statistical analysis

The data will be analysed with SPSS 22.0 software (IBM Corp, Armonk, NY, USA). Two analyses will be performed based on the IPA classification, one in which patients with possible IPA will be excluded in the calculation and a second analysis in which patients with possible IPA will be classified as not having IPA. Therefore, the comparisons and analyses will be (1) proven/probable IPA versus no IPA, and (2) proven/probable IPA versus possible/no IPA [43]. The sensitivity, specificity, negative predictive value (NPV), positive predictive value (PPV), and diagnostic odds ratio, including 95% confidence intervals, will be calculated for different biomarkers. ROC analysis will be performed and AUC (including 95% CI) and cut-off values will be established from the ROC curves for GM, RT-PCR, TAFC and bmGT. The McNemar χ^2^ test will be used to compare sensitivity, specificity, NPV, and PPV between different assays. The difference in hospital stay and the survival between possible or no IPA versus proven or probable IPA will be analysed with chi-square tests, and the relationships between the levels of biomarkers and the hospital stay and survival will be analysed via regression models. *P* < 0.05 will be defined as statistically significant.

### Results of pilot study

Sputa used for spiking tests were obtained from four patients who were not diagnosed with IPA. TAFC and bmGT were undetectable in these samples. The detected concentrations of the 5 ng/mL and 40 ng/mL TAFC and bmGT standards spiked into the sputa from the four no IPA patients are presented in Table 4. The method was found to be accurate and precise and has high recovery for both TAFC and bmGT from spiked sputum samples (Table 4). The accuracy (%deviation), precision (%RSD) and recovery (%) with our protocol for TAFC/bmGT detection at a 5 ng/mL level were ≤ ±5.0/5.0%, 5.2/7.3% and 98.4/96.9%, respectively.

**Table 4:** Evaluation of accuracy, precision, and recovery of TAFC and bmGT from spiked sputum samples (mean ± SD)

Spiked TAFC & bmGT (ng/mL)	Detected TAFC (%deviation)	Detected bmGT (%deviation)	TAFC Recovery % (%RSD)	bmGT Recovery % (%RSD)
0	Not detected	Not detected	Not applicable	Not applicable
5	4.9 ± 0.3 (−1.6)	4.8 ± 0.4 (−3.1)	98.4 ± 0.1 (5.2)	96.9 ± 0.1 (7.3)
40	38.7 ± 1.2 (−3.3)	39.0 ± 2.9 (−2.5)	96.7 ± 3.0 (3.1)	97.5 ± 7.3 (7.4)

**Fig. 3:**
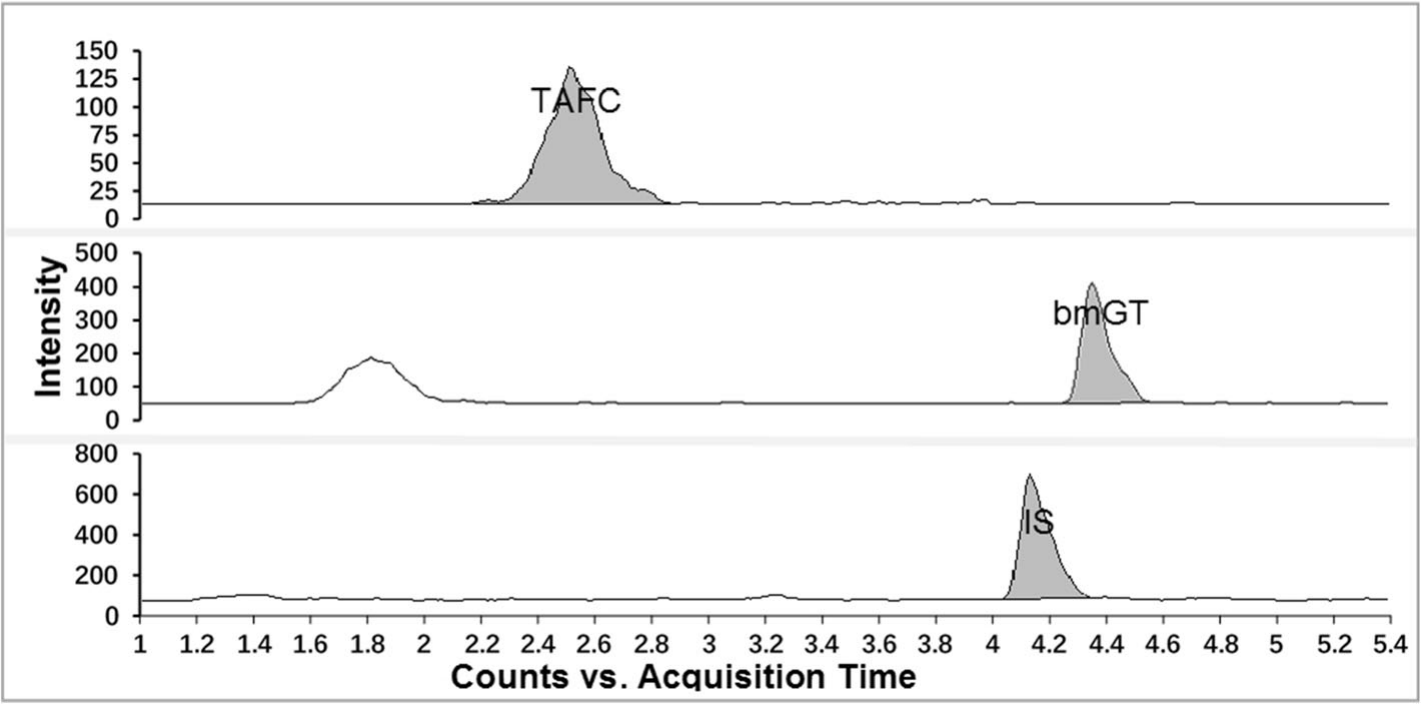
HPLC-MS/MS detection of TAFC and bmGT in the sputum of a probable IPA patient. Abbreviations: bmGT = bis(methylthio)gliotoxin; TAFC = triacetylfusarinine; IS = internal standard (phenacetin)

Sputum culture for the patient with lung cancer and probable IPA yielded *Aspergillus fumigatus* twice while the serum GM showed negative result (ODI = 0.12). TAFC and bmGT were detectable in this patient’s sputum and the concentrations are 45.36 ng/mL and 46.35 ng/mL, respectively (Fig. 3).

## Discussion

The clinical and radiological features of IPA are atypical, leading to the difficulty in an early diagnosis and treatment of the disease. There are still uncertainties and controversies associated with the diagnosis of IPA in clinical practice. Intensive efforts have been made to improve the diagnosis in “high-risk” individuals such as haematological and immunocompromised patients. Consequently, a series of approaches have been developed attempting to access a precise diagnosis.

Biomarker development has opened new possibilities in the diagnosis of a great number of disorders including IPA. Biomarkers related to IPA have been isolated and investigated largely in serum or BALF sample, yet few study has addressed the possibility of sputum biomarkers in the diagnosis of IPA. Each sample type has its advantages as well as disadvantages (Table 5). Biomarker detection using serum is convenient, inexpensive and time-saving, presenting high specificity [20], however the sensitivity was reported to be low in non-haematological patients [6, 23, 44, 45]. In comparison, BALF assays exhibit higher sensitivity as well as specificity in both neutropenic [46] and non-neutropenic patients [7, 9], probably because BALF is retrieved from local airways. However, the bronchoscopy is an invasive and costly procedure, and it might not be accessible to severe patients or those with contraindications.

**Table 5:** Pros and cons of serum, BALF and sputum samples for IPA diagnosis in non-haematological patients

Samples	Pros	Cons
Serum	□Convenient, cheap and fast □High specificity	□Low sensitivity
BALF	□Local-infection related □High sensitivity and specificity	□Invasive, having contraindications □Expensive
Sputum	□Convenient, cheap and fast □Local-infection related □Non-invasive	□Affected by colonization and contamination

Sputum is the phlegm from the respiratory tract that has altered macromolecular, polymer composition and biophysical properties varying with diseases. Studies investigating sputum biomarkers are often limited by the techniques to obtain the specimen; however, intensive studies have demonstrated that hypertonic saline-induced sputum can be processed to yield reproducible results that reflect disease activity and correlate with BALF [47]. *Aspergillus*-related biomarkers, including *A*-DNA and GM, have been investigated using sputum samples, and we have developed a method to detect TAFC and bmGT in sputum; therefore, it is technically possible to use sputum for biomarker detection in the diagnosis of IPA.

Although no studies have investigated the superiority of the sputum assay over serum or BALF for the diagnosis of IPA in non-haematological patients, the sputum assay might be a valuable and useful approach for the detection of *Aspergillus* infection in patients with pre-existing respiratory diseases due to the following reasons. First, non-neutropenic patients tend to develop airway invasive forms of IPA [24, 25], which implies that airway-retrieved samples might be more sensitive to *Aspergillus* constituents. Additionally, Escribano et al. [48] identified matching genotypes of *Aspergillus fumigatus* in BALF and the sputum/bronchial secretion samples in proven or probable IPA patients, which indicates a comparable accuracy of BALF and sputum detection for the diagnosis of IPA. Furthermore, in the study by Kimura et al. [27], sputum GM was elevated in all patients with proven or probable IPA, and sputum GM showed a higher concentration (average GM index of 4.5) than that in BALF and serum (1.4 and 1.0, respectively). The above evidence strongly supports the usefulness of the sputum assay for the diagnosis of IPA.

Potential diagnostic biomarkers for IPA have been intensively studied, given the problem of an early diagnosis. We have reviewed the literature systematically to identify potential biomarkers that can be detected in a sputum sample. Galactomannan (GM) is a cell wall polysaccharide that is released by *Aspergillus* during fungal growth. The method of GM detection has been commercialized and widely used in the clinic with serum and BALF samples [19]. The serum GM assay exhibits low sensitivity (23.1–42%) for the diagnosis of IPA in non-haematological patients [6, 23], whereas BALF GM shows higher sensitivity and specificity [6–9]. The GM assay for sputum samples has been established in several studies (Table 1). Whether a GM assay based on lower airway derived sputum can be used as a non-invasive tool for the diagnosis of IPA deserves investigation. The measurement of GM in sputum might provide a more sensitive and non-invasive diagnostic approach for IPA.

GM detection can be affected by non-*Aspergillus* fungi and food that contains GM and beta-lactam antibiotics, which can produce false-positive values [49–51]. Several other biomarkers have been shown to play a role in the diagnosis of IPA and may be potential candidates for future clinical use. A rapid detection with LFD is an immunochromatographic assay that uses a monoclonal antibody called JF5 that targets an extracellular glycoprotein secreted during active fungal growth [36]. The test can be performed bedside and accomplished within 15 mins and, therefore, can be used as an early indicator for *Aspergillus* infection. The device is highly specific to antigens from *Aspergillus* but does not react with antigens from a large number of clinically important fungi, especially *Candida species* [36]. Pan et al. [52] conducted a meta-analysis to investigate its diagnostic accuracy for invasive aspergillosis, and showed that the LFD assay using BALF had higher sensitivity (86%) and specificity (93%) than serum (68% sensitivity and 87% specificity). Importantly, the BALF LFD assay also exhibited satisfying diagnostic performance for IPA (77% sensitivity and 92% specificity) in patients with URD [11]. Sputum supernatant has a similar matrix as BALF after processing procedures; hence, the use of the sputum LFD assay may provide a bedside and time-saving diagnostic tool for IPA.

TAFC is a siderophore secreted soon after conidiospore germination by *Aspergillus* in iron-limited media and is essential for fungal virulence [53, 54]. In a rat model of *Aspergillus fumigatus* infection, (68)Ga radiolabelled *Aspergillus* TAFC selectively accumulates in infected lung tissue [55]. Recently, Carrol et al. [37] detected TAFC in the serum of patients at risk for invasive aspergillosis using ultra-performance liquid chromatography tandem mass spectrometry. TAFC might be a potential early diagnostic biomarker for IPA. Gliotoxin (GT) is the major and the most potent toxin produced by *Aspergillus fumigatus* and mediates immunosuppressive and proapoptotic effects that may promote the establishment of invasive aspergillosis [56–58]; however, the rapid elimination of GT from body fluids by adjoining tissues and circulating cells precludes its feasibility as a biomarker for IPA [59–61]. bmGT is the inactive metabolite derived from GT and is quite stable after secreted, therefore it has been studied for the possibility of being an alternative biomarker [61]. Matxalen et al. [62] found that serum bmGT presented higher sensitivity (61.5%) and similar specificity (93%) compared to serum GM in haematological patients for the diagnosis of IPA. The assays for TAFC and bmGT have not yet been developed and validated in sputum. After a series of strict experiments, we first identified and standardized the TAFC and bmGT assays in sputum via the HPLC-MS/MS technique. We further validated the methods by determining the sputa induced from a group of patients with probable IPA or no IPA, which is consistent with the clinical diagnosis showing the great potential of being a non-invasive and useful marker for IPA. The detailed methods to process sputum and detect TAFC and bmGT in sputum have been submitted for patents (State Intellectual Property Office in China, No.201710428772.9). In this study, the diagnostic performance of sputum TAFC and bmGT assays for the diagnosis of IPA in patients with underlying respiratory disease will first be examined.

RT-PCR is a precise approach to detect *A*-DNA, and it has been applied for serum and BALF samples even though there is a lack of standardized protocol. Several meta-analyses [21, 63–67] assessed the efficacy of PCR assays on these two sample types for the diagnosis of IPA in haematological patients. The results showed that BALF PCR had much higher sensitivity (approximately 90%) and specificity (> 90%) than serum (85% for sensitivity and 75% for specificity). Several studies [28, 29, 68, 69] used PCR to identify *Aspergillus* in sputum and showed that sputum PCR had higher sensitivity than routine fungal culture; however, this technique has not been applied in the diagnosis of IPA and will be addressed in our study.

Sputum is a type of sample that is viscous and heterogeneous. To accurately detect biomarkers in sputum plugs, a proper processing method is needed. Dithiothreitol (DDT) is a commonly used reagent to homogenize sputum before all kinds of constituents are examined, and it is recommended for the pre-treatment of viscous samples by guidelines [70]. Baxter et al. [28] processed sputum with DDT and sonication before performing a GM assay and the results showed excellent GM reproducibility and low interassay and intraassay coefficients of variation. However, Prattes et al. [71] examined the effect of DDT on a BALF GM assay and found conflicting results. GM levels in BALF were substantially decreased from 0.51 ODI to 0.01 ODI after pre-treatment with DTT, and the LFD assay in BALF was also dramatically affected. Although this study targeted BALF, it is likely that DTT might affect sputum GM and LFD detection. Therefore, to minimize the effect of DTT on these biomarker assays, we will pre-treat sputum samples with phosphate buffered saline, which is in accordance with the method by Kimura et al. [27].

There are concerns about the specificity of the sputum assay related to colonization or contamination. Nevertheless, the four biomarkers (GM, JF5 antigen, TAFC, bmGT) in our study are produced and released during fungal growth. It is less likely that the increase in the levels of these biomarkers is a result of contamination of fungal spores. In addition, lower airway secretions will be used by sputum induction, and sputum quality control by squamous cell counting and viability calculation will further exclude the possibility of contamination. We will also determine an appropriate cut-off value of different sputum biomarkers, which will help to differentiate colonization from infection.

There are several highlights of this study. We systematically evaluate the validity of various biomarkers based on sputum from lower airways for the diagnosis of IPA for the first time; in particular, the establishment of a method that simultaneously detects TAFC and bmGT in sputum is first described and applied for patents and reported. Furthermore, the study population, i.e., patients with URD, has been increasingly recognized to present with IPA, which is associated with high mortality [12], but only has been addressed in a small number of studies. We assess the sputum biomarkers for IPA diagnosis in this population for the first time, and the results may help to provide novel, non-invasive and valuable diagnostic tools for IPA in patients with URD. In addition to sputum, serum and BALF will also be collected and compared for their diagnostic performance. In the statistical analysis, we will conduct two different analyses to investigate the impact of the possible IPA group on the interpretation of the results. Except for the investigation of diagnostic efficacy, we will also conduct a clinical follow-up to observe the prognosis of different types of IPA and further investigate host factors predisposing to IPA in patients with URD.

This study has some limitations that should be considered. The categories of probable and possible IPA are proposed for immunocompromised patients only in the consensus report by the EORTC/MSG. However, this report recognizes that the revised definition of IPA does not necessarily apply to immunocompetent patients as it is difficult to find a sufficient basis for identifying appropriate host factors. Patients with URD may possess much different risk factors to *Aspergillus* infection compared to those immunocompromised individuals. As in the study by Prattes et al. [11], pre-existing respiratory disease is added as an independent host factor to establish the diagnosis in our study. We will also conduct post hoc analyses of the host factors linked to diagnosis and prognosis for further investigation. The concentration of biomarkers in the sputum may fluctuate with the production and expectoration of the sputum and the releasing of the biomarkers from the infection site. However, this study already includes a great number of biomarkers from different sample types with a comparable large sample size, and we do not perform serial tests for sputum biomarkers at different time points across the disease course, which may lead to the underestimation of the diagnostic performance of several biomarkers. Further study may be warranted to investigate the dynamic variation of different *Aspergillus* biomarkers in sputum.

In conclusion, the design and protocol of a clinical trial that investigates the performance of sputum signatures for the diagnosis and prognosis of IPA in patients with URD are presented here. The results of this innovative study are expected to provide answers to the following questions. Are sputum signatures valuable diagnostic tools for IPA in patients with URD? Which signature is the strongest potential candidate? Would the modified host factors be appropriate for this patient population? What disease course of IPA would present in this population? The study findings will provide evidence and novel insights into these areas, which have not been accessed.

## Abbreviations

A-DNA: Aspergillus DNA
AUC: Area under the curve
BALF: Bronchoalveolar lavage fluid
bmGT: Bis(methylthio)gliotoxin
COPD: Chronic obstructive pulmonary disease
DDT: Dithiothreitol
EORTC/MSG: European Organization for Research and Treatment of Cancer/Mycoses Study Group
GM: Galactomannan
HPLC-MS/MS: High-performance liquid chromatography tandem mass spectrometry
IPA: Invasive pulmonary aspergillosis
LFD: Lateral flow device
NPV: Negative predictive value
ODI: Optical density index
PCR: Polymerase chain reaction
PPV: Positive predictive value
ROC: Receiver operating characteristics
RT-PCR: Real time polymerase chain reaction
SPARED: Sputum signatures for invasive pulmonary aspergillosis in patients with underlying respiratory diseases
TAFC: Triacetylfusarinine
URD: Underlying respiratory diseases
